# Identifying Obstetric Mistreatment Experiences in U.S. Birth Narratives: Application of Internationally Informed Mistreatment Typologies

**DOI:** 10.1097/NMC.0000000000000811

**Published:** 2022-04-27

**Authors:** Hannah J. Tello, Dylan J. Téllez, Joseph E. Gonzales

**Affiliations:** **Dr. Hannah J. Tello** is Health Data Analyst, Greater Lowell Health Alliance, Department of Psychology, University of Massachusetts Lowell, Lowell, MA. Dr. Tello can be reached via email at HTello@greaterlowellhealthalliance.org; **Dr. Dylan J. Téllez** is an Instructor, Department of Psychology, University of Massachusetts Lowell, Lowell, MA.; **Dr. Joseph E. Gonzales** is an Assistant Professor, Department of Psychology, Center for Women and Work, & Center for Health Statistics, University of Massachusetts Lowell, Lowell, MA.

**Keywords:** Labor, Maternal–child nursing, Obstetric, Obstetric nursing, Parturition, Pregnancy, Psychological trauma

## Abstract

**Background:**

Traumatic births are those resulting in feelings of distress that persist after the birth experience. Health care providers may play a role in these experiences through various forms of mistreatment. Analyses of global birth experiences have generated several domains of mistreatment. This study applies these evidence-based domains of mistreatment as an a priori coding scheme for analysis of 96 oral narratives of U.S.-based births to describe the nature of perceived mistreatment using participants' own descriptions of experiences.

**Method:**

Ninety-six transcripts of oral birth stories from 61 participants were coded using the domains of mistreatment experiences described by the Bohren et al.'s (2015) systematic review of obstetric mistreatment.

**Results:**

*N* = 131 individual experiences of perceived obstetric mistreatment were identified in 41 out of 96 narratives (42.7%). The most frequent types of experiences were Poor Rapport (90 incidences) and Failure to Meet Professional Standards of Care (29).

**Clinical Implications:**

Although most women in our study did not perceive any instances of obstetric mistreatment during their childbirth, over 40% of participants noted at least one event that fit one of the typologies we used as a framework for analysis. Visibility and review of the types of perceived mistreatment experiences that occur during birth enables health system leaders to implement prevention and accountability strategies. Most instances of perceived mistreatment during birth may be prevented through intentional implementation of individualized, respectful, supportive care during labor and birth.

## Abstract 2

Women may experience a tramatic birth when they perceive they have not been respected, listened to, or treated kindly during the childbirth process. Procedures and protocols that are routine to nurses, midwives, and physicians are not always understood or desired by women giving birth. In this study, narratives of women who gave birth in the United States reveal more effort is needed to make sure all those who experience childbirth are treated with the respect they deserve.

**Figure:**
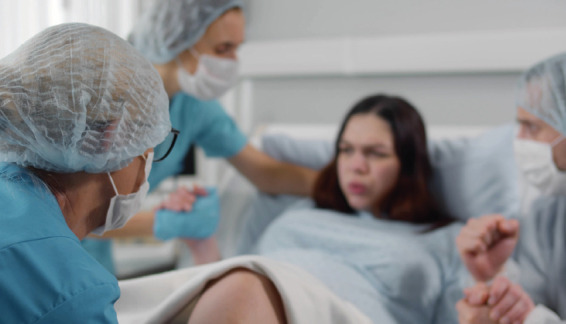
No caption available.

Obstetric mistreatment, dehumanizing treatment by medical personnel that results in an experience of maternal victimization via the loss of autonomy, is a factor contributing to traumatic birth experiences (Darilek, 2018). The World Health Organization (WHO) has acknowledged the global prevalence of these experiences and the need to mitigate them, asserting that people in labor are entitled to “the highest attainable standard of health, which includes the right to dignified, respectful health care” (WHO, 2014, p. 1) and recommending “respectful maternity care... [that] maintains [women's] dignity, privacy and confidentiality, and ensures freedom from harm and mistreatment [during birth]” (WHO, 2018, p. 3) as the global standard of care. This global call to action is supported by literature on maternal perceptions of mistreatment, though most of these inquiries are based on birth experiences outside the United States (Allen, 1998; Moyzakitis, 2004; Nicholls & Ayers, 2007; Thomson & Downe, 2008), suggesting a need for investigations into the phenomenon of perceived mistreatment in U.S.-based birth experiences.

One of the most robust assessments of domains of obstetric mistreatment was conducted by Bohren et al. (2015). Researchers applied a thematic synthesis approach across 65 studies on experiences of mistreatment during birth from 34 countries, generating an evidence-based classification system of perinatal mistreatment experiences consisting of seven domains: physical abuse, verbal abuse, sexual abuse, stigma and discrimination, professional care failure, poor rapport, and system conditions. A strength of the study is its use of a systematic review yielding an evidence-based typology describing domains of mistreatment that can be applied in future research to identify frequencies and types of mistreatment across populations. Although the study incorporated international experiences, it may not be representative of any specific country. Only one study from the United States met inclusion criteria, compared with, for example, 11 studies based in sub-Saharan Africa, and five each from Asia, the Middle East and North Africa, and Latin America.

Development of an evidence-based typology laid the groundwork for application of the domains in further research. One of the U.S.-based expansions is Vedam et al.'s (2019) application of the mistreatment domains in a 60-item survey as part of the *Giving Voice to Mothers* project. In their sample of 2,138 U.S. births, 17.3% of participants self-reported experiencing some form of mistreatment in accordance with the original domains, suggesting the value of the domains as a framework for assessing mistreatment in U.S. births in other contexts and through other research approaches.

One limitation of survey-based applications of the domains as an assessment strategy is that this approach relies on participant identification of their experiences matching Vedam et al.'s (2019) item wording, which can overly constrain instances in which participant experiences meet domain criteria. In contrast, this is a strength of a priori coding methodology, where experiential criteria are identified directly from participants' descriptions of their birth experiences (Blair, 2015). Other qualitative approaches have been applied successfully to U.S. births, using inductive thematic coding (Reed et al., 2017), phenomenological approaches (Beck, 2004), and meta-ethnographies reciprocally analyzed to identify shared themes (Elmir et al., 2010).

In our study, we extend on Bohren et al.'s (2015) and Vedam et al.'s (2019) work in two ways to describe the topography of obstetric mistreatment in a U.S. sample when applying internationally informed obstetric mistreatment domains. First, we apply Bohren et al.'s seven domains of obstetric mistreatment (Poor Rapport, Failure to Meet Professional Standards of Care, Stigma and Discrimination, Verbal Abuse, System Conditions, Physical and Sexual Abuse) as a coding methodology to a U.S. sample of oral birth stories. These original domains were identified using a thematic synthesis approach and therefore represent broad, descriptive themes that describe types of mistreatment phenomena observed across qualitative findings from multiple studies. This domain identification approach used by Bohren et al. is distinct from a review or evaluation of medical records against standards of care to identify objective failures to meet stated care standards identified by licensing or other governing bodies, an approach that would be especially difficult to use given the heterogeneity of the original data sample, which includes studies from multiple countries, each with their own systems for oversight of care standards. Application of these themes as descriptive categories is therefore appropriate for use in the analysis of narratives, in which descriptions of experiences are the primary data unit. Rather than using a closed-form assessment method (Vedam et al., 2019), we apply the domains to the analysis of oral narratives where evaluative linguistic indicators (e.g., *It was traumatic*) and affective indicators (e.g., crying during narration) are used to interpret meaning from the participants' own descriptions and perceptions of their experiences during birth to determine whether Bohren et al.'s domain criteria are met.

## Methods

### Participants

Participants over age 18 who had given birth in the United States were recruited via paper and digital flyers. The call for participants read *Participate in a research study on birth and infant feeding. If you are at least 18 years old and have experience giving birth, then we want you to interview for a study on your experience*. Participants were not required to have experienced obstetric mistreatment to participate. Although birth, postpartum, and infant feeding experiences were collected for this sample, only birth narratives are analyzed in this study. Paper copies of the flyer were distributed to three pediatric offices, and other public spaces (e.g., college campuses, and community message boards) within the New England region of the United States. Digital copies of the flyer were distributed via national maternal health professional ListServs, email contact lists for parenting and infant feeding groups, and via a local provider network contact list of approximately 300 clinical providers for distribution to their networks and patients, and for inclusion in their newsletters or social media pages. Recruitment was open from June 2018 through January 2020. This sample size is three times larger than the recommendation for thematic saturation (Hennink et al., 2017) and was selected to protect against under identification of anticipated low-frequency domain events (e.g., Sexual Abuse).

### Measures

#### Preinterview Online Survey

The survey gathered participants' demographic information (e.g., birth history, age, race and ethnicity, marital status, and residency). The survey intake collected additional information not used in the current study (e.g., mode and experience of infant feeding).

#### Interview Protocol

The interview protocol elicited narrative responses about participants' experiences with pregnancy, birth, and the postpartum period; there were no questions specific to mistreatment, victimization, or care quality. During the interview, participants were asked to share their birth story *in detail, as [they] experienced it, including any relevant circumstances leading up the birth that [they] consider important*. Participants were instructed to include *any memory that feels especially vivid or meaningful, including anything that [they] were thinking or feeling at the time*. Participants with multiple birth experiences were invited to share their stories in any order that made the most sense to them.

### Procedure

Consent was obtained from participants before completion of the preinterview online survey and at the beginning of their interview. All interviews were audio-recorded for later transcription. Participants were read a debriefing protocol at the end of their interview that summarized the research process, provided contact information for the research team, and offered local and national resources for any participant interested in support after recollecting negative experiences. The narratives were recorded and promptly transcribed with all identifying information (e.g., names and locations) removed. Upon transcription, completion original audio files were deleted. All study materials and protocols were reviewed and approved by our Institutional Review Board.

### Analysis

Audio recordings were initially transcribed using Transcribe (Wreally, 2019) and checked for transcription accuracy by researchers. Transcripts were coded in NVivo 12 (QSR, 2018) using the seven obstetric mistreatment domains identified by Bohren et al. (2015). To meet the domain criteria, Bohren et al.'s first-order identification criteria of specific event types were used. For example, the domain of Poor Rapport includes the second-order theme of “ineffective communication,” which includes the criteria of poor communication, dismissal of women's concerns, language and interpretation issues, and poor staff attitudes.

We relied on participants' own description of their birth experiences as the primary unit of analysis; therefore, researchers did not correct or edit participant descriptions even when they were likely to contain misapplied or inaccurate medical terminology. Instead, to focus coding on the perceptual experiences of participants and mitigate potential effects of coder bias, incidents were only coded when 1) they met one of Bohren et al.'s (2015) domain criteria and 2) additional evaluative or affective indicators reflected that the participant perceived the incident as mistreatment. Identification of evaluative indicators was guided by the Appraisal Framework (Martin & White, 2005), which describes linguistic features that indicate attitudinal assessments in narratives.

For example, a participant said, *I had a panic attack. I was not really aware of certain aspects of the surgery... It was traumatic*. In this example, *I was not really aware of certain aspects of the surgery* met the domain criteria for Poor Rapport (primary domain) via ineffective communication (second-order theme) according to the identifying criteria of poor communication (first-order theme criteria); *it was traumatic* is an evaluative statement reflecting the participants' assessment of their experience, and therefore meets the coding criteria. Similarly, *Nobody responded and nobody did anything [cries]* met the domain criteria for Poor Rapport, and the indicator of *cries* satisfies the paralinguistic criteria. In contrast, a participant stated, *[The physician] just wasn't paying attention to us. But for the most part I was fine with that*. *[The physician] just wasn't paying any attention to us* met the criteria for Poor Rapport, but the participants' own evaluation (*I was fine with that*) indicates no perceived mistreatment, and therefore this was not coded as Poor Rapport.

## Results

### Participant Demographics

Sixty-one participants contributed 96 oral birth narratives; some participants reported more than one birth experience. Average participant age was 34 years old (*SD* = 4.9; Table 1). Most were married (82%), followed by those in a committed partnership (13.1%), and those not in a relationship (4.9%). Participants mostly identified as white (88.5%), followed by Asian/Asian-American (3.3%), Black or African American (3.3%), Middle Eastern (1.6%), and Biracial (1.6%); 6.6% of participants were Hispanic or Latina. Birth experiences include vaginal birth without induction (37.5%), induced vaginal birth (25%), scheduled cesarean birth (15.6%), and emergency cesarean birth (19.8%). Most participants were from the Northeast (60.7%), followed by the Midwest (13.1%), South (13.1%), and West (6.6%). The median time since birth was 2 years with an interquartile range of 1 to 6 years.

**TABLE 1.** PARTICIPANT DEMOGRAPHIC DATA

Characteristic	Descriptive Statistics
**Age**	
Mean (SD)	34.3 (4.1)
**Time Since Birth (in years)**	
Mean (SD)	4.6 (8.6)
Median	2
25% to 75% Percentile	1-6
**Characteristic**	***n* (%)**
**Partnership Status**	
Married	50 (82.0)
Committed Partnership	8 (13.1)
Not in Relationship	3 (4.9)
**Race/Ethnicity**	
White	54 (88.5)
Black or African American	2 (3.3)
Asian or Asian American	2 (3.3)
Middle Eastern	1 (1.6)
Biracial	1 (1.6)
Hispanic or Latina	1 (1.6)
**Births per Participant**	
One	31 (32.3)
Two	23 (24.0)
Three	6 (6.3)
Four	1 (1.0)
**Birth Mode**	
Vaginal Birth, No Induction of Labor	36 (37.5)
Vaginal Birth, Labor Induced	24 (25.0)
Cesarean Birth, Scheduled	15 (15.6)
Cesarean Birth, Emergency	19 (19.8)

### Identification of Bohren et al.'s (2015) Obstetric Mistreatment Domains

Two coders reviewed narratives with 60% overlap across codes. Coders' interrater reliability was estimated using Cohen's Kappa and indicated strong agreement (κ = 0.801). Fifty-five narratives (57.3%) did not include any instances of mistreatment. The other 41 (42.7%) narratives had an average of 3.11 (*SD* = 3.04) unique instances per narrative, totaling 131 unique instances from 37 unique participants in our sample. The most frequently identified domain was Poor Rapport (68.7% of all mistreatment codes), followed by Failure to Meet Professional Standards of Care (22.1%), Stigma and Discrimination (6.9%), Verbal Abuse (1.5%), and System Conditions (.8%); there were no instances of Physical and Sexual Abuse identified.

#### Poor Rapport Domain

**Ineffective communication**. Poor communication, dismissal of maternal concerns, and language and interpretation issues met the criteria for ineffective communication, totaling 71 incidences (Table 2). Poor communication between participants and their providers contributed to participant distress. Some experienced anxiety at seeing providers react to emergency situations without understanding the circumstances; one participant described her distress at seeing her physician *freaking out* while another recalled her provider *acting in a panic*. Some participants responded to perceived distress among their health care team with their own panic; one recalled, *And all of a sudden I had nurses coming in... I had no idea what was going on but people are reaching [all around] and I just started bawling*. Another recalled feeling upset by lack of information about circumstances surrounding her emergency cesarean stating, *There was a lot of like motion and commotion and like sort of an emergency feel to the whole thing...And I was just like, ‘Wait, what?’...It was upsetting and traumatic*. Participants who didn't feel like procedures were explained to them felt unsupported by their providers.

**TABLE 2.** POOR RAPPORT DOMAIN SUBCATEGORY EXAMPLES AND FREQUENCIES

Second-Order Themes	Example in Current Data	Frequency
Ineffective Communication	*I just started bawling...I did- and I was just really upset about the whole situation, and I guess I would have liked to know what was happening before hand or while they were doing it*.	
	*I felt like I didn't really know what was going on. .I was- just like I feel like I don't know what's happening. I feel like nobody's really communicating with me*.	71
	*I told them I was like, I still feel everything on the right side of my body, and they kept telling me that's how the epidural works is it starts on one side. It's like bilateral and it will eventually like work and it took me - it took hours and it took me like crying for them to finally realize that like the epidural wasn't (pause) done correctly*.	
Lack of Supportive Care	*I literally felt like a cold piece of meat like laying on a slab of like metal because that's pretty much what was going on. I was freezing*.	
	*[During my cesarean] I said, ‘Excuse me, I really feel like I'm going to throw up’ and nobody responded, and nobody did anything and no – nobody was looking at me (cries). Not my partner, not the anesthesiologist ... nobody responded and nobody looked at me*.	8
Loss of Autonomy	*During that time I had to fight for my right to get out of the bed and walk around*.	
	*I basically had to ask the nurse- like repeatedly if I can sort of get onto my hands and knees on the bed in order to help the pushing because I felt like I would be in a better position and better able to push her down that way. They were pretty resistant to it, which I found really frustrating... I wasn't completely numb and I also was very comfortable with how I was able to move and I was not planning on getting off the bed to do this but they wouldn't let me*.	11
	*I had no idea what to do so I was just doing whatever my doctors told me to do and I felt violated*.	
	TOTAL	90

Some participants reported that their concerns were disregarded. A participant recalled, *I kept saying like – people aren't listening to me. People aren't hearing me, people are- you know- it's oh she's just another mom*. Some mothers also felt like their pain was dismissed, with one recalling, *It took me like crying for them to finally realize that [the epidural] wasn't done correctly*. Dismissing maternal concerns contributed to maternal distress, with one participant summarizing, *I was a just angry and hurt and scared*.

**Loss of autonomy**. Objectification, lack of respect for preferred birth positions and practices, denial of food and mobility, and treating participants as passive participants in their births met the criteria for loss of autonomy, totaling 11 incidences. Some participants felt mistreated after having their mobility restricted in ways they felt were unnecessary. One participant described *having to fight for [her] right to move around*; another participant remarked, *I found it really frustrating [being restricted] ... [the nurses] were just so resistant...like I had to force them [to let me change positions]*. Some participants spoke broadly in terms of their loss of control of their births. For example, a participant summarized, *I was just getting dragged along by all like these people and just – I had no control...I felt very emotionally just crushed*.

**Lack of supportive care**. Experiences in which participants felt poorly treated due to care that lacked compassion, courtesy, or respect met the criteria for lack of supportive care, totaling 8 incidences. Participants felt dehumanized by routine procedures. One participant described feeling *like a cold piece of meat* immediately following her cesarean. Another noted a difference in support between her nurses and her doctor regarding cervical checks, *The nurses were really great. But the doctors who would come in - this was like routine for them. So they were not very gentle. They're just like, ‘Oh just checking another woman.’ So like that was awful*. Participants felt unsupported by providers, even when they were physically present; one recalled, *[During my cesarean] I said, ‘Excuse me, I really feel like I'm going to throw up’ and nobody responded, and nobody did anything - nobody was looking at me [cries]*.

#### Failure to Meet Professional Standards of Care Domain

**Exams and procedures**. Physical exams and procedures that are regarded as typical courses of care sometimes resulted in perceived mistreatment from participants, totaling 12 incidences (Table 3). Inductions were a common source of maternal distress in this category, due to both physical and psychological impacts. One participant described their induction as *torture*; another noted that their induction made them feel *totally out of control*. Some participants attributed the pain of their procedures directly to their care being rough or aggressive; one participant recalled her water breaking during a routine exam, prior to the start of labor, stating, *It was pretty forced from the doctor*. In one particularly significant incident, a participant recalled her experience of failed pain management during her cesarean birth, summarizing, *After the spinal [block] - it wasn't tested and I had a C-section with no pain medication and so I was screaming that I could feel everything like very like traumatic to your whole body right? As well as your brain [cries]*.

**TABLE 3.** FAILURE TO MEET PROFESSIONAL STANDARDS OF CARE DOMAIN SUBCATEGORY EXAMPLES AND FREQUENCIES

Second-Order Themes	Example in Current Data	Frequency
Lack of Informed Consent and Confidentiality	*[The doctor] (pause) gave me pitocin and I said that I didn't want pitocin and he told - he patted my hand and said, “Well you lost your right to refuse when you came here.” ... He told me I lost my like right to refuse*.	
	*No one's really telling me what's going on. They're just telling me what to do. And the doctor comes in... And literally just inserts his fingers inside of my vagina to check me without any preamble at all (pause). And then luckily my baby dad's mom was there, Grandma, and she had to almost forcibly remove the doctor for me because I started panicking ... None of it was okay*.	9
Physical Exams and Procedures	*After the spinal [block] it wasn't tested and I had a c-section with no pain medication and so I was screaming that I could feel everything like very like traumatic to your whole body right? As well as your brain [cries]*.	
	*My water broke during the exam on the table. Um so I mean I think it was pretty forced from the doctor*.	12
	*I was like why not just give me the meds that do work? Why- why do this to me? There was a ton of bleeding from it. It was awful*.	
Neglect and Abandonment	*I was definitely suffering. And my midwife wasn't really there, she kind of left me alone, that became a source of a lot of sort of feelings of abandonment for me, you know*.	
	*So they left me [in the hospital room] for two days. [I knew] something's not right ... needless to say [after two days] it turned out to be an emergency C-section ... it was horrible. It was traumatizing*.	8
	TOTAL	29

**Lack of informed consent**. Some participants reported that they had not provided informed consent for procedures, totaling 9 incidences. Cervical checks were a common procedure associated with perceived mistreatment due to lack of consent. One participant recalled, *The doctor comes in and literally just inserts his fingers inside of my vagina to check me without any preamble at all [pause]...I started panicking*. Another participant disagreed with her provider about whether she was informed about a cervical check, stating, *[She claimed] she said, ‘I'm going to check you.’ I didn't hear that at all. Next thing you know I'm being checked...and I freaked. I screamed and yelled and jerked*.

Although some experiences of lack of informed consent processes were associated with perceived pressure or coercion to comply (e.g., *I kept saying numerous times that I did not want [a Foley] ...more doctors would come in and pressure me*), other experiences were more explicit. One participant was told she had lost her right to refuse procedures: *I said that I didn't want Pitocin and he told - he patted my hand and said, ‘Well you lost your right to refuse when you came [to the hospital]*. Some participants noted that, upon reflection, they had had the right to limit procedures, but felt pressured to comply at the time. One participant commented, *And I realized after I had every right [to decline] legally but I was presented like I had no options, like I couldn't. It was horrible [cries]*.

**Neglect and abandonment**. Provider neglect or long delays met the criteria for neglect and abandonment, totaling 8 incidences. One participant reported continued distress over feeling abandoned, stating, *I was definitely suffering. And my midwife wasn't really there. That became a source of a lot of sort of feelings of abandonment*. Prolonged inductions were also a source of distress in this category. One participant recalled, *So they left me [in the hospital room] for two days. [I knew] something's not right ... it was horrible. It was traumatizing*. One participant recalled the distress of having her repeated efforts to get the attention of her providers fail, stating, *I thought I was going to die at that point. I was [in] so much pain for so long...I don't understand why nobody came to me, like, why are you ignoring me?*

#### Stigma and Discrimination Domain Narratives

**Discrimination based on sociodemographic factors**. Criteria were met for instances of discrimination based on age, socioeconomic status, and marital status, totaling 7 incidences (Table 4). Some participants felt their care quality was reduced due to stigma of being teen parents. A participant summarized, *I feel as though they were just dealing with an 18-year-old, and they did not want to talk to me. I didn't rate on their radar*. Other participants linked their perceived poorer care to their socioeconomic status, with one summarizing, *I don't think [middle class] women get treated that way so that was pretty horrible to realize ... that if you're perceived to be poor, you get different care*.

**TABLE 4.** STIGMA AND DISCRIMINATION DOMAIN SUBCATEGORY EXAMPLES AND FREQUENCIES

Second-Order Themes	Example in Current Data	Frequency
Based on Sociodemographics	*I feel as though they were just dealing with an 18 year old and they did not want to talk to me. I didn't rate on their radar of a mother who could understand so they just didn't bother*.	
	*Because the way I was treated was 100% unacceptable and I don't think [middle class] women get treated that way so that was pretty horrible to realize ... that if you're perceived to be poor you get different care*.	7
Based on Medical Conditions	*They are so worried but you know all my tests, my actual urine tests were negative right and it's like, Okay I understand you have to worry about this and you see a fat woman and you assume there's a problem but how bout you look at the numbers, the actual tests, and not stereo type, like- this isn't evidence based medicine*.	
	*The thing that disturbed me the most was I had signs on my door entering where I was ... for all these precautions because of the herpes. Like everybody was afraid they were going to get it...They came in- the nursing staff would come in clothed head to toe coverage like with a mask and the funny little Johnny thing and little booties on their feet and you know gloves up because I guess they thought I was going to give it to them ... But so I was just upset*.	2
	TOTAL	9

**Discrimination based on medical conditions**. Two participants experienced perceived mistreatment due to their health conditions. One participant felt discriminated against due to her herpes diagnosis, stating, *The thing that disturbed me the most was I had signs on my door for all these precautions. Like everybody was afraid they were going to get it. ... so I was just upset*. Another participant felt her care quality was diminished due to her weight, remarking, *[They] see a fat woman [and] assume... and stereotype, like- this isn't evidence-based medicine*. In these instances, participants noted that this discrimination negatively impacted their trust in their providers.

#### Verbal Abuse Domain

**Harsh language**. Only one instance of rude language met the domain criteria (Table 5). A participant recalled a provider who made several remarks about her daughter's anticipated size, *He made a lot of really jerky comments. He called her an elephant at some point while ... he was rude*.

**TABLE 5.** VERBAL ABUSE DOMAIN SUBCATEGORY EXAMPLES AND FREQUENCIES

Second-Order Themes	Example in Current Data	Frequency
Harsh Language	*He made a lot of really jerky comments ... he called her an elephant at some point while ... he was rude ... he was not my favorite person at all*.	1
Threats and Blaming	*So rather than just like shutting off the pitocin the nurse came in to me and she was like - and I'm never going to forget- She said, “So what? Are you going to be a wuss and wuss out on us and have a C-section or are you not just going to tough it out to see if he can give birth correctly?” Never forget that*.	1
	TOTAL	2

**Threats and blaming**. One participant recalled abusive language and blame from her provider regarding choosing a cesarean, stating, *[The nurse] was like- and I'm never going to forget this- she said, ‘So what? Are you going to wuss out on us and have a C-section or are you just going to tough it out to see if you can give birth correctly?’ Never forget that*.

#### System Conditions Domain

**Lack of resources**. One participant experienced distress over the perceived lack of resources at her hospital (Table 6). She recalled not being able to use a shower due to a monitor not being available, summarizing, *The pain is progressing... then they finally get me a unit. Nine hours later. It was ridiculous*.

**TABLE 6:** SYSTEM CONDITIONS DOMAIN SUBCATEGORY EXAMPLES AND FREQUENCIES

Second-Order Themes	Example in Current Data	Frequency
Lack of Resources	*And they kept saying they didn't have one available. So while the pain is progressing to the point that hot water is no longer going to help because I haven't been in it then they finally get me a ...ng unit. Nine hours Later. It was ridiculous*.	1
	TOTAL	1

## Discussion

Although most women in our study did not perceive any instances of obstetric mistreatment during their childbirth, over 40% of participants noted at least one event that fit one of the typologies we used as a framework for analysis. The methodological approach used in this study is an internationally informed series of obstetric mistreatment domains first presented by Bohren et al. (2015) applied as a priori codes in a qualitative analysis of U.S-based oral birth stories. We identified five of Bohren et al.'s seven domains of obstetric mistreatment in our sample of U.S. birth narratives. There were no instances of Physical or Sexual Abuse identified in this sample of oral narratives, suggesting that these categories may describe the phenomena of perceived mistreatment in global samples, but are less prevalent in U.S. birth experiences. Systems Conditions were similarly infrequent in this sample, which is likely due to the current study's focus on birth experiences, whereas most of the subcategory items (i.e., billing issues) in System Conditions would be present in postpartum settings. Researchers who use the original typology to assess birth experience should consider this in their methodology and analysis.

Our study also reported few instances of Stigma and Discrimination. Given the known frequency of these experiences in U.S. birth experiences (Attanasio & Hardeman, 2019; Davis, 2019), this is likely a reflection of the coding standard applied in our study, which would require a participant to explicitly link, through evaluative statements, their mistreatment experience to a specific experience of stigma or discrimination to meet the coding criteria. Studies seeking to describe the nature of discrimination as a form of mistreatment during birth should consider a different coding or methodological approach to accurately capture the scope of these experiences.

Using Bohren et al.'s (2015) internationally informed typology of domains as an a priori coding scheme contributes to both our understanding of the nature of mistreatment in the United States, as well as provides a novel strategy for analyzing birth narratives. Our study required presence of either evaluative or affective indicators participants' distress to meet coding criteria, which may alleviate methodological concerns regarding coder bias in narrative data projects. Ability to apply a range of strategies to identify mistreatment is an additional benefit of using oral narrative, in particular, in which paralinguistic indicators can be used.

### Limitations

One potential limitation of our study is the unknown impact of time since birth experience on participants' recall. We were interested in participants' perceptions of their birth experiences. Thus, our research methodology was not designed to address questions concerning the veracity of recollections. Some evidence suggests time-from-event memory decay results in less accurate recollections (Lacy & Stark, 2013), whereas some does not (Diamond et al., 2020). Although it is possible that the recall quality of these significant life events may decay over time, there is evidence that affective recall quality is reliable despite the passage of time (Porter & Birt, 2001).

Our participants were predominantly white (88%) and our results are limited in understanding of U.S. birth experiences of underrepresented minority groups. In the United States, the disproportionately high rate of maternal and infant mortality for Black mothers (Krisberg, 2019) and the disproportionate risk of Black women experiencing medical discrimination (Attanasio & Hardeman, 2019; Vedam et al., 2019) are urgent health priorities. Studies using phenomenological methods to identify instances of obstetric racism are especially useful in this area (Davis, 2019). Vedam et al. (2019) also used the Bohren et al. (2015) domains as the framework for their survey study, which included a more representative proportion of Black participants, and found that it captured obstetric mistreatment experiences of racial minority mothers, suggesting the Bohren at al. domains could be an appropriate methodology in more representative samples of birth narratives.

## Clinical Nursing Implications

Understanding perceptions of mistreatment in birth experiences supports development of evidence-based practices for nursing practice. Interactions with providers are reported to be common factors driving trauma after birth (Reed et al., 2017). This is an opportunity to elevate the high-stakes role of health care providers, particularly nurses, as potential mediators of perceived mistreatment during birth. Perceptions of women that they were not treated with respect or listened to during the childbirth process are evidence that maternity care needs improvement. In the United States, nurses provide the majority of hands-on care during childbirth (National Academies of Sciences, Engineering, and Medicine, 2020), thus nurses are positioned to make sure each woman receives respectful maternity care.

Our findings are further evidence that U.S. births are at risk of including an experience of perceived obstetric mistreatment. Many of the instances of perceived mistreatment may be prevented with relatively simple mitigation strategies. Most of the codes for mistreatment were within the domain of Poor Rapport. Some participants indicated that feeling their concerns were heard or being given the opportunity to connect with their providers would have improved their experiences, with one summarizing, *I wish that people would have listened to me*. Participants felt that simple changes in communication, like more communication in advance of procedures, may have prevented their distress. Nurses can facilitate clear, compassionate communication on shared decision-making as a useful tool for preventing perceived mistreatment within this domain. More robust interventions to address systemic change may consider evidence-based quality improvement protocols used in the context of identification, prevention, and response to perceived mistreatment experiences.

Our study contributes to the growing body of research on maternal perceptions of mistreatment during their birth experiences, as well as offers a framework for analyzing oral narratives as a valuable source of data. Research that centers the lived experiences of those who have labored and given birth is critical for ensuring that the future development of evidence-based best practices prioritizes the mitigation of potential harm and psychological distress from perceived mistreatment.

### Acknowledgment

This research was supported in part by grant R50180000038539 from the College of Fine Arts, Humanities and Social Sciences and the Office of the Vice Chancellor for Research and Innovation at the University of Massachusetts Lowell (Joseph E. Gonzales, PI, and Robin Toof, Ainat Koren, and Hannah J. Tello, Co-PIs).

## SUGGESTED APPLICATION FOR CLINICAL NURSING PRACTICE

- Everyone who gives birth deserves respectful maternity care.
- What physicians, midwives, and nurses consider routine care and usual procedures may not be perceived as such by patients during labor and birth.
- Listening to women, hearing their concerns, acting on them, answering their questions fully, explaining procedures before they are done, and giving them options, are ways to promote respectful maternity care.
- Clinical education for physicians, midwives, and nurses, and health care facility education programs should emphasize that a lack of intent to cause harm is not sufficient to prevent maternal distress or trauma as the perception of mistreatment plays an essential role in patient experience.
- Care settings must cultivate an environment of accountability in which nurses are empowered to identify, interrupt, and report mistreatment without retaliation.
- Most mistreatment experiences may be prevented with relatively simple measures that center supportive care practices, individualized care, and communication with people in labor.
- Systematic or policy drivers of experiences of perceived mistreatment during birth should be reviewed and amended as a prevention measure (i.e., policies that delay access to pain relief, or that result in frequent personnel turnover resulting in patients working with unknown care providers).
- Vulnerability to mistreatment is not equally distributed; patients who are Black, Hispanic, uninsured, and others are at higher risk of mistreatment. Where disparities in outcomes are present, the role of mistreatment should be considered as an especially critical driver to be interrupted and corrected.
